# Development and validation of a nomogram to predict overall survival for patients with metastatic renal cell carcinoma

**DOI:** 10.1186/s12885-020-07586-7

**Published:** 2020-11-04

**Authors:** Wenwen Zheng, Weiwei Zhu, Shengqiang Yu, Kangqi Li, Yuexia Ding, Qingna Wu, Qiling Tang, Quan Zhao, Congxiao Lu, Chenyu Guo

**Affiliations:** 1 Department of Education, Yantai Yuhuangding Hospital, Qingdao University, Yantai, China; 2 Drug Clinical Trial Agency, Yantai Yuhuangding Hospital, Qingdao University, Yantai, China; 3 Department of Urology, Yantai Yuhuangding Hospital, Qingdao University, Yantai, China; 4 Department of Pharmacy, Yantai Yuhuangding Hospital, Qingdao University, No.20, Yuhuangdingdong Road, Yantai, Shandong China

**Keywords:** Metastatic renal cell carcinoma, SEER, Overall survival, Prognosis, Nomogram

## Abstract

**Background:**

Heterogeneity of metastatic renal cell carcinoma (RCC) constraints accurate prognosis prediction of the tumor. We therefore aimed at developing a novel nomogram for accurate prediction of overall survival (OS) of patients with metastatic RCC.

**Methods:**

We extracted 2010 to 2016 data for metastatic RCC patients in the Surveillance, Epidemiology, and End Results (SEER) database, and randomly stratified them equally into training and validation sets. Prognostic factors for OS were analyzed using Cox regression models, and thereafter integrated into a 1, 3 and 5-year OS predictive nomogram. The nomogram was validated using the training and validation sets. The performance of this model was evaluated by the Harrell’s concordance index (C-index), calibration curve, integrated discrimination improvement (IDI), category-free net reclassification improvement (NRI), index of prediction accuracy (IPA), and decision curve analysis (DCA).

**Results:**

Overall, 2315 metastatic RCC patients in the SEER database who fulfilled our inclusion criteria were utilized in constructing a nomogram for predicting OS of newly diagnosed metastatic RCC patients. The nomogram incorporated eight clinical factors: Fuhrman grade, lymph node status, sarcomatoid feature, cancer-directed surgery and bone, brain, liver, and lung metastases, all significantly associated with OS. The model was superior to the American Joint Committee on Cancer (AJCC) staging system (7th edition) both in training (C-indices, 0.701 vs. 0.612, *P* < 0.001) and validation sets (C-indices, 0.676 vs. 0.600, *P* < 0.001). The calibration plots of the nomogram corresponded well between predicted and observed values. NRI, IDI, and IPA further validated the superior predictive capability of the nomogram relative to the AJCC staging system. The DCA plots revealed reliable clinical application of our model in prognosis prediction of metastatic RCC patients.

**Conclusions:**

We developed and validated an accurate nomogram for individual OS prediction of metastatic RCC patients. This nomogram can be applied in design of clinical trials, patient counseling, and rationalizing therapeutic modalities.

## Background

Kidney cancer is one of the most prevalent genitourinary malignancies. In 2018 alone, it accounted for 2.2% of new cancer cases and 1.8% of overall cancer deaths worldwide [1]. In the United States alone, new cases and deaths due to kidney and renal pelvis cancer in 2019 were 73,820 and 14,770, respectively [2]. RCC is the most common subtype of kidney neoplasms, accounting for 90–95% of these cancers [3]. Based on the SEER database, 16% of kidney cancer patients presented with metastatic tumors at diagnosis. Also, the 5-year survival rate of metastatic kidney cancer patients was only 13.0% during the 2010–2016 period [4]. The national Swedish kidney cancer register reported comparable findings, in which between 2005 and 2010, 15–23% of the RCC patients presented with metastatic forms of the disease at diagnosis [5].

Accurate prognostic models are invaluable in designing clinical trials, patient psychological management, and governing therapeutic modalities. Currently, the Memorial Sloan-Kettering Cancer Center (MSKCC) and the International Metastatic RCC Database Consortium (IMDC) models are the most widely used prognostic models for metastatic RCC. Karnofsky performance, level of serum lactate dehydrogenase, hemoglobin, and corrected calcium as well as time from diagnosis to initiation of treatment are the independent prognostic factors in the MSKCC model [6]. The efficiency of the MSKCC model was validated in an independent study of 353 participants, previously untreated for metastatic RCC [7]. In the era of targeted therapy, Heng et al. identified 645 patients with metastatic RCC treated with sunitinib, sorafenib, or bevacizumab plus interferon from the IMDC during 2004–2008, and proposed the IMDC model including Karnofsky performance status, serum hemoglobin, corrected serum calcium, time from diagnosis to treatment together with neutrophils and platelets [8]. The two models provide the median OS for each group after stratifying metastatic RCC patients based on the prognostic risk factors.

Clinical and pathological characteristics of metastatic RCC patients are highly heterogeneous. In addition, they exhibit highly variable survival time [9]. Besides the MSKCC and IMDC models, individual survival prediction can also be implemented by risk-scoring systems. Unfortunately, best to our knowledge, there is no risk-scoring system for metastatic RCC patients. Accordingly, we aimed at constructing a predictive nomogram for patients newly diagnosed with metastatic RCC by combining clinical and pathological characteristics derived from the SEER database. In addition, we further aimed at evaluating the discrimination, calibration, and clinical use of this model.

## Methods

### Data source

We used data in the publicly available SEER database (http://www.seer.cancer.gov), which covers approximately 28% of the US population. SEER provides data on patient demographics, primary tumor site, tumor stage, surgical treatment, patient survival among others. Relevant data was retrieved using SEER*Stat software (version 8.3.5).

### Study population

**Fig. 1:**
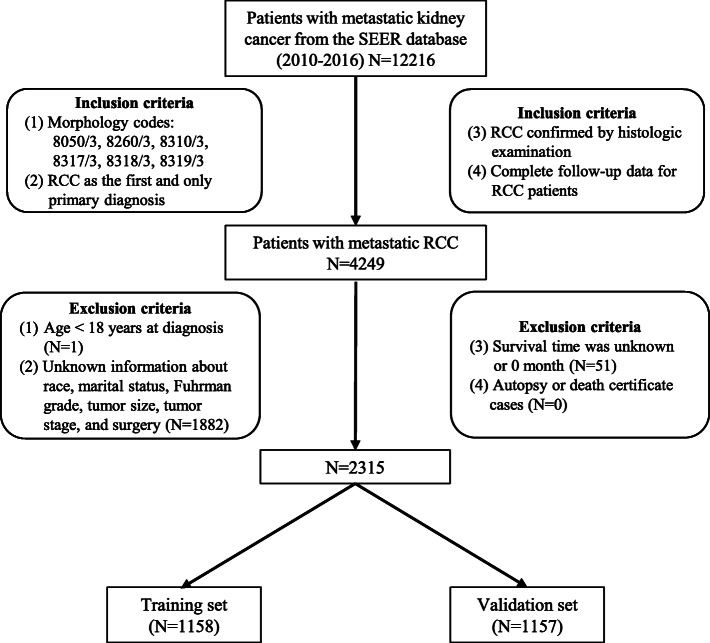
Diagrammatic flow of patient selection

To be included in this study, participants must have first presented with metastasis RCC at diagnosis. The RCC must have been identified using universal morphology codes (8050/3, 8260/3, 8310/3, 8317/3, 8318/3, and 8319/3) based on the International Classification of Diseases for Oncology codes (3rd edition). Besides, the RCC must have been the first and only primary disease. The diagnoses were confirmed by histological examination and there was complete follow-up data of the patients. Patients under 18 years at diagnosis, missing data on follow-up, race, marital status, Fuhrman grade, tumor size, tumor stage, lymph node status, metastasis, and surgery were all excluded from the study. Autopsy or death certificate cases were also excluded. In the end, 2315 metastatic RCC patients fulfilled the inclusion criteria, and were included in the final analyses. The flow diagram for patient selection is presented in Fig. 1.

### Measurable variables

Demographic and clinical variables such as age at diagnosis, race (black, white, other), sex, marital status (married, unmarried), histologic subtype (clear cell renal cell carcinoma (CCRCC), papillary renal cell carcinoma (PRCC), chromophobe renal cell carcinoma (CHRCC), sarcomatoid renal cell carcinoma (SRCC), collecting duct renal cell carcinoma (CDRCC)), Fuhrman grade (grade I, grade II, grade III, grade IV), Tumor classification (T1, T2, T3, T4, TX), lymph node status (N0, N1, NX), sarcomatoid feature (yes, no, unknown), cancer-directed surgery (recommended and performed, recommended but not performed, not recommended), bone, brain, liver, and lung metastasis status, survival time and vital status were all captured. The AJCC Cancer Staging Manual (7th edition, 2010) was employed to evaluate tumor stages.

### Ascertainment of the outcome

OS was the primary outcome of this study, defined as survival time between metastatic RCC diagnoses to death, attributed to any cause. OS was ascertained based on the code “vital status” in the SEER database.

### Statistical analysis

Selected patients were randomly and equally stratified into training and validation sets. Preliminary descriptive statistics were performed to describe the baseline characteristics of the patients in both sets. Thereafter, normally distributed continuous variables were expressed as mean ± the standard deviation, whereas non-normal continuous variables were presented by medians (interquartile range). Categorical variables were summarized in frequencies and percentages. Univariable and multivariable Cox regression analyses were performed on the training set to obtain crude and adjusted hazard ratios (HRs), important in identifying prognostic factors significant for OS. Prognostic factors were determined by a backward stepwise process using the Bayesian information criterion. Schoenfeld residuals were used to assess the proportional hazards assumption of Cox regression models.

Nomograms are pictorial representations that quantify risks and the probability of clinical events by scoring the involved factors. They have been demonstrated to generate more precise predictions than the conventional AJCC staging system in several types of cancers [10, 11]. In this study, a nomogram for predicting 1, 3 and 5-year OS was derived based on the findings of multivariable Cox regression analysis.

Discrimination and calibration, important properties in evaluating the performance of the model [12, 13], were both assessed in our study. C-index was applied to evaluate the discriminative ability of the nomogram, which depicted the probability of the predicted risk was higher for a random patient having an event than for a random patient not having an event. After comparing the predicted probability of events for all possible pairs of patients, C-index is 0.5 if the model can not discriminate the patients with and without events. Conversely, C-index is 1 if the probability predicted by the model is always higher for patients with events than those without events [14]. The robust performance of the model was assessed using the original and optimism-corrected C-indices. Calibration plot, the best visual representation of the relationship between predicted risk and actual risk, was presented using bootstrap resampling method [14]. Calibration plots fall on a 45-degree diagonal line, reflecting excellent absolute risk estimates. NRI and IDI usually assess and quantify the refinement in risk prediction between new and old models [15]. NRI is based on reclassification tables composed of patients with and without events, and can quantify the correct reclassification in categories. NRI is calculated by adding the percentage of patients with events who are correctly reclassified to the percentage of patients without events who are correctly reclassified [14]. IDI reflects the improvement in sensitivity and specificity of a model. It is also an integrated difference in Youden’s indices [15]. IDI is calculated by adding the increased probability predicted by new model compared to old model for patients with events to the decreased probability predicted by new model compared to old model for patients without events [15]. Therefore, NRI and IDI were both employed to compare the discriminative ability between the new model and the AJCC staging system. Notably, even though NRI and IDI have become increasingly popular, they should be interpreted with caution [16]. IPA is a promising metric that combines discrimination and calibration in one value, thus improves interpretability by adjusting for the benchmark model [17, 18]. IPA was also reported in this study to reflect the performance of the model. DCA is a method for evaluating the benefits of a diagnostic test across a range of patient preferences for accepting risk of undertreatment and overtreatment to facilitate decisions about test selection and use [19]. Unlike the sensitivity, specificity, and area under the curve, DCA directly assesses the utility of clinical risk prediction models for decision making [20]. Herein, DCA was plotted to evaluate the clinical value of the nomogram by quantifying the net benefit in comparison with the AJCC staging system.

Statistical tests were performed using R software (version 3.5.2, http://www.r-project.org/). All tests were two-sided, with statistical significance set at *P* < 0.05.

## Results

### Patient baseline characteristics

Data for 2315 eligible metastatic RCC patients collected between 2010 and 2016 were extracted from the SEER database, and then randomly stratified equally into two sets, with 1158 and 1157 patients in the training and validation sets, respectively. The median age of the participants was 61 (54–68) years. Whites accounted for 83.8% of the patients. With regard to gender, 1629 (70.4%) patients were males. The majority of the patients (67.3%) were married. The most common histologic subtype was CCRCC (84.6%), with Grade III (40.0%) being the most common Fuhrman grade. The majority of the tumors were T3 (56.8%), with T2 and T4 accounting for the rest (16.5 and 12.7%, respectively). Concerning treatment, 85.6% of the patients underwent cancer-directed surgery. Overall, at diagnosis, 31.5, 9.9, 11.0, and 61.7% of the patients presented with bone, brain, liver, and lung metastases, respectively.

The median follow-up time of the cohort was 16 months, whereas the median OS time was 19 months (95% CI, 18–21 months). The median follow-up time for patients without events was 30 months. By the end of the survey, 1563 patients had died, of which 1467 from RCC and 96 from other causes. The demographic and clinical characteristics of the patients were summarized in Table 1.

**Table 1:** Demographics and clinical characteristics for the metastatic RCC patients

Variables	All patients (***n*** = 2315)	Training set (***n*** = 1158)	Validation set (***n*** = 1157)
	No. (%)	No. (%)	No. (%)
**Age (years)**	61 (54–68)	60 (54–68)	61 (54–68)
**Race**			
White	1941 (83.8%)	968 (83.6%)	973 (84.1%)
Black	174 (7.5%)	92 (7.9%)	82 (7.1%)
Other	200 (8.6%)	98 (8.5%)	102 (8.8%)
**Sex**			
Male	1629 (70.4%)	812 (70.1%)	817 (70.6%)
Female	686 (29.6%)	346 (29.9%)	340 (29.4%)
**Marital status**			
Married	1559 (67.3%)	777 (67.1%)	782 (67.6%)
Unmarried	756 (32.7%)	381 (32.9%)	375 (32.4%)
**Histologic subtype**			
CCRCC	1959 (84.6%)	982 (84.8%)	977 (84.4%)
PRCC	142 (6.1%)	56 (4.8%)	86 (7.4%)
CHRCC	27 (1.2%)	14 (1.2%)	13 (1.1%)
SRCC	168 (7.3%)	91 (7.9%)	77 (6.7%)
CDRCC	19 (0.8%)	15 (1.3%)	4 (0.3%)
**Fuhrman grade**			
Grade I	57 (2.5%)	26 (2.2%)	31 (2.7%)
Grade II	516 (22.3%)	247 (21.3%)	269 (23.2%)
Grade III	926 (40.0%)	482 (41.6%)	444 (38.4%)
Grade IV	816 (35.2%)	403 (34.8%)	413 (35.7%)
**Tumor size (mm)**	90 (70–118)	90.5 (70–120)	90 (68–115)
**Tumor classification**			
T1	289 (12.5%)	141 (12.2%)	148 (12.8%)
T2	383 (16.5%)	198 (17.1%)	185 (16.0%)
T3	1316 (56.8%)	661 (57.1%)	655 (56.6%)
T4	293 (12.7%)	143 (12.3%)	150 (13.0%)
TX	34 (1.5%)	15 (1.3%)	19 (1.6%)
**Lymph node status**			
N0	1618 (69.9%)	796 (68.7%)	822 (71.0%)
N1	597 (25.8%)	310 (26.8%)	287 (24.8%)
NX	100 (4.3%)	52 (4.5%)	48 (4.1%)
**Sarcomatoid feature**			
Yes	446 (19.3%)	220 (19.0%)	226 (19.5%)
No	1697 (73.3%)	848 (73.2%)	849 (73.4%)
Unknown	172 (7.4%)	90 (7.8%)	82 (7.1%)
**Cancer-directed surgery**			
Recommended and performed	1982 (85.6%)	980 (84.6%)	1002 (86.6%)
Recommended but not performed	21 (0.9%)	12 (1.1%)	9 (0.6%)
Not recommended	312 (13.5%)	166 (14.3%)	146 (12.8%)
**Bone metastasis**			
Yes	729 (31.5%)	361 (31.2%)	368 (31.8%)
No	1586 (68.5%)	797 (68.8%)	789 (68.2%)
**Brain metastasis**			
Yes	230 (9.9%)	112 (9.7%)	118 (10.2%)
No	2085 (90.1%)	1046 (90.3%)	1039 (89.8%)
**Liver metastasis**			
Yes	254 (11.0%)	126 (10.9%)	128 (11.1%)
No	2061 (89.0%)	1032 (89.1%)	1029 (88.9%)
**Lung metastasis**			
Yes	1429 (61.7%)	723 (62.4%)	706 (61.0%)
No	886 (38.3%)	435 (37.6%)	451 (39.0%)

*Abbreviation*: *CCRCC* clear cell renal cell carcinoma, *PRCC* papillary renal cell carcinoma, *CHRCC* chromophobe renal cell carcinoma, *SRCC* sarcomatoid renal cell carcinoma, *CDRCC* collecting duct renal cell carcinoma

### Independent prognostic factors

Univariable and multivariable Cox regression analyses were performed to explore independent risk factors for OS. Crude and adjusted HRs were presented in Table 2. After adjustment for other risk factors, identified eight variables: Fuhrman grade, lymph node status, sarcomatoid feature, cancer-directed surgery, bone, brain, liver, and lung metastases were significantly associated with OS. Subgroup analyses (Table 3) also showed patients not recommended for nephrectomy were more likely to succumb to death than those who underwent nephrectomy, indicating the robustness of the model.

**Table 2:** Univariable and multivariable Cox regression analysis of OS for metastatic RCC patients

Variables	Univariable analysis		Multivariable analysis	
	HR (95% CI)	***P***	HR (95% CI)	***P***
**Age**	1.01 (1.00–1.02)	0.025		
**Race**				
White	Ref.			
Black	1.26 (0.99–1.61)	0.06		
Other	1.08 (0.84–1.38)	0.561		
**Sex**				
Male	Ref.			
Female	1.07 (0.92–1.24)	0.383		
**Marital status**				
Married	Ref.			
Unmarried	1.12 (0.97–1.30)	0.117		
**Histologic subtype**				
CCRCC	Ref.			
PRCC	1.28 (0.95–1.74)	0.106		
CHRCC	1.06 (0.55–2.04)	0.874		
SRCC	2.25 (1.78–2.85)	< 0.001		
CDRCC	2.37 (1.40–4.03)	0.001		
**Fuhrman grade**				
Grade I	Ref.		Ref.	
Grade II	0.81 (0.48–1.35)	0.411	1.04 (0.62–1.76)	0.876
Grade III	0.94 (0.57–1.55)	0.809	1.34 (0.80–2.25)	0.268
Grade IV	1.46 (0.88–2.41)	0.142	1.73 (1.02–2.95)	0.042
**Tumor size**	1.001 (1.000–1.002)	0.002		
**Tumor classification**				
T1	Ref.			
T2	1.42 (1.08–1.87)	0.013		
T3	1.47 (1.16–1.86)	0.002		
T4	2.43 (1.83–3.23)	< 0.001		
TX	5.63 (3.18–9.97)	< 0.001		
**Lymph node status**				
N0	Ref.		Ref.	
N1	1.87 (1.61–2.18)	< 0.001	1.55 (1.32–1.81)	< 0.001
NX	1.85 (1.35–2.53)	< 0.001	1.62 (1.17–2.25)	0.004
**Sarcomatoid feature**				
Yes	Ref.		Ref.	
No	0.47 (0.40–0.56)	< 0.001	0.61 (0.50–0.74)	< 0.001
Unknown	1.05 (0.80–1.38)	0.709	0.80 (0.58–1.11)	0.175
**Cancer-directed surgery**				
Recommended and performed	Ref.		Ref.	
Recommended but not performed	4.37 (2.46–7.76)	< 0.001	4.15 (2.25–7.66)	< 0.001
Not recommended	2.54 (2.12–3.04)	< 0.001	2.30 (1.85–2.86)	< 0.001
**Bone metastasis**				
Yes	Ref.		Ref.	
No	0.80 (0.69–0.93)	0.003	0.68 (0.58–0.79)	< 0.001
**Brain metastasis**				
Yes	Ref.		Ref.	
No	0.54 (0.44–0.68)	< 0.001	0.60 (0.48–0.76)	< 0.001
**Liver metastasis**				
Yes	Ref.		Ref.	
No	0.54 (0.44–0.66)	< 0.001	0.66 (0.54–0.82)	< 0.001
**Lung metastasis**				
Yes	Ref.		Ref.	
No	0.65 (0.56–0.75)	< 0.001	0.64 (0.55–0.75)	< 0.001

*Abbreviation*: *HR* hazard ratio, *95% CI* 95% confidence interval, *CCRCC* clear cell renal cell carcinoma, *PRCC* papillary renal cell carcinoma, *CHRCC* chromophobe renal cell carcinoma, *SRCC* sarcomatoid renal cell carcinoma, *CDRCC* collecting duct renal cell carcinoma

**Table 3:** Subgroup analyses for patients who underwent or did not undergo nephrectomy

Subgroups	HR (95% CI) ^**a**^	***P***
**Fuhrman grade**		
Grade I	3.23 (1.22–8.53)	0.018
Grade II	2.04 (1.51–2.75)	< 0.001
Grade III	3.48 (2.75–4.41)	< 0.001
Grade IV	1.53 (1.06–2.23)	0.024
**Lymph node status**		
N0	2.94 (2.38–3.64)	< 0.001
N1	1.71 (1.30–2.25)	< 0.001
NX	3.03 (1.71–5.35)	< 0.001
**Sarcomatoid feature**		
Yes	2.01 (1.25–3.26)	0.005
No	2.40 (1.99–2.89)	< 0.001
Unknown	3.13 (1.95–5.04)	< 0.001
**Bone metastasis**		
Yes	2.46 (1.87–3.26)	< 0.001
No	2.43 (2.00–2.95)	< 0.001
**Brain metastasis**		
Yes	2.16 (1.43–3.25)	< 0.001
No	2.46 (2.07–2.94)	< 0.001
**Liver metastasis**		
Yes	3.09 (2.05–4.65)	< 0.001
sNo	2.36 (1.99–2.81)	< 0.001
**Lung metastasis**		
Yes	2.50 (2.08–3.01)	< 0.001
No	2.22 (1.62–3.06)	< 0.001

^a^: Nephrectomy not recommended vs. nephrectomy recommended and performed

*Abbreviation*: *HR* hazard ratio, *95% CI* 95% confidence interval

### Nomogram construction

**Fig. 2:**
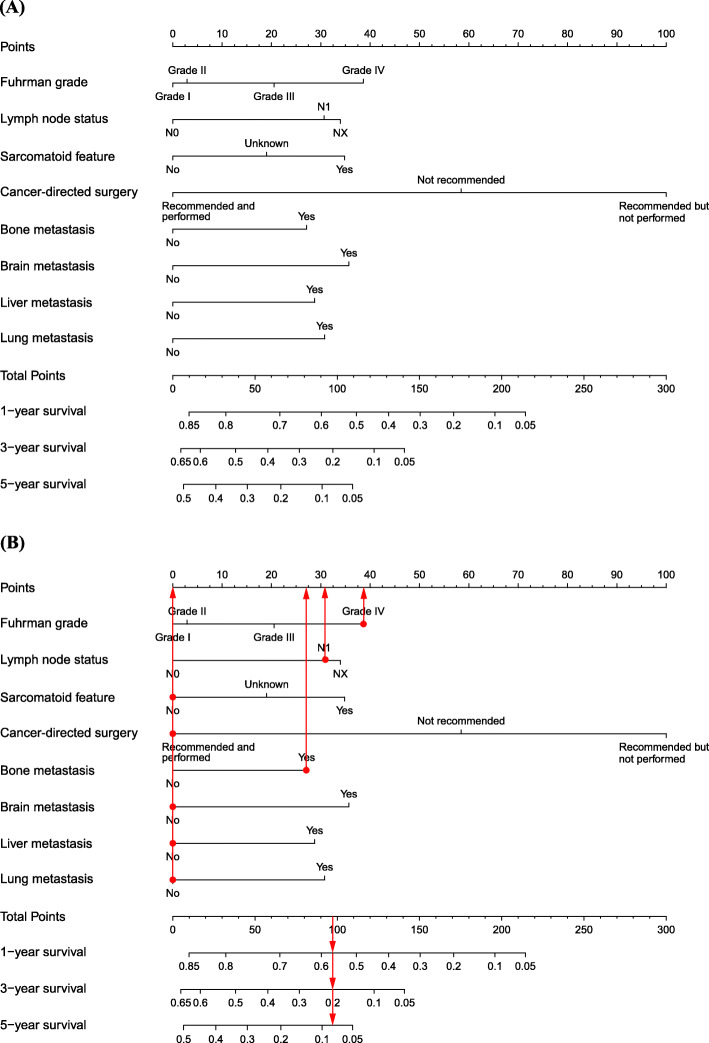
Nomogram for 1, 3, and 5-year prediction of OS of patients with metastasis RCC **a** Classical application of the nomogram **b**. Each category of the prognostic variables was assigned a score on the Points scale. After summing up the score of each variable and locating the total score on the Total Points scale, a line was vertically drawn to the 1, 3, and 5-year survival probability scale and estimated survival probability at each time point could be obtained

The 1, 3 and 5-year predictive nomogram for OS were constructed only incorporating the significant prognostic factors. As shown in the nomogram (Fig. 2), cancer-directed surgery was the most significant factor for prognosis, followed by Fuhrman grade, brain metastasis, and sarcomatoid feature. Lymph node status, lung, and liver metastases moderately impacted on OS. Meanwhile, Bone metastasis had the least effect on OS.

### Classical application of the nomogram

The practical use of the nomogram was presented in Fig. 2. Here, a patient was diagnosed with Fuhrman grade IV, N1 stage metastatic RCC. He lacked sarcomatoid differentiation and displayed no bone metastasis. After assessment, he underwent nephrectomy. The nomogram scored 39 points for Fuhrman grade IV, 31 for N1 stage, 0 for no sarcomatoid feature, 27 for bone metastasis, and 0 for cancer-directed surgery, totaling 97 points. This corresponded with a 1, 3 and 5-year survival probability of 57, 20, and 8%, respectively.

### Nomogram performance

**Fig. 3:**
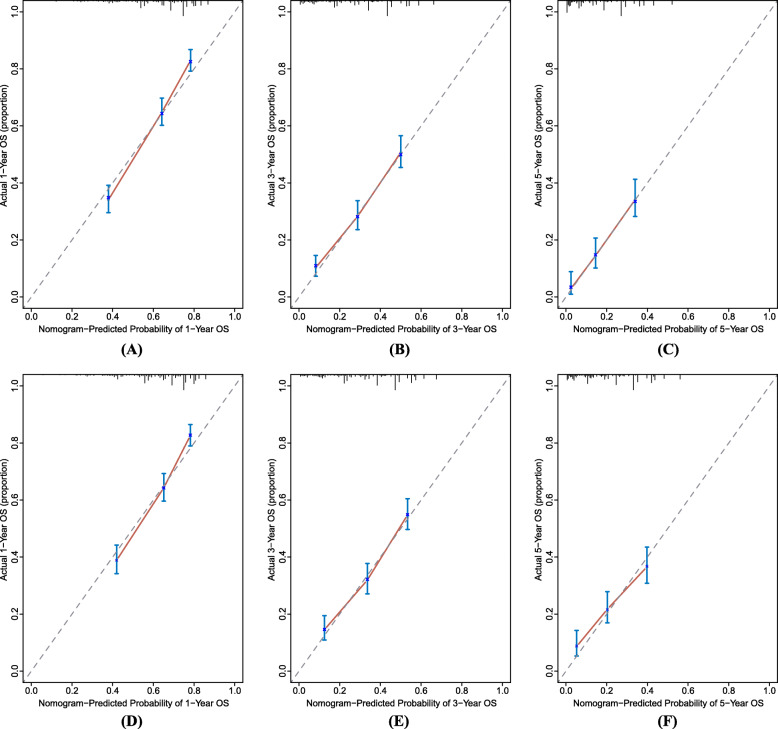
Calibration curves for predicting 1, 3 and 5-year OS of patients with metastasis RCC in training set **a**, **b**, **c** and validation set **d**, **e**, **f**. The nomogram-predicted probability of OS was plotted on the x-axis, with actual OS plotted on the y-axis. The calibration curves were visual representations of the relationship between the predicted and actual absolute risk

Discrimination and calibration were employed to evaluate the performance of the model. The C-indices of the nomogram were 0.701 (95% CI, 0.682–0.720) for the training set and 0.676 (95% CI, 0.655–0.697) for the validation set. With respect to the AJCC staging system, the C-indices were 0.612 (95% CI, 0.591–0.633) for the training set and 0.600 (95% CI, 0.578–0.622) for the validation set, significantly lower than those of the nomogram (*P* < 0.001). Across the 1000 bootstrap resamples, the optimism-corrected C-indices of the nomogram were 0.698 (95% CI: 0.677–0.717) and 0.677 (95% CI: 0.656–0.697) for the training and validation sets, respectively, indicative of robustness in the performance of the model. Calibrations of the nomogram were assessed using actual and predicted estimates after bootstrapping with 1000 resamples. Calibration plots (Fig. 3) revealed good consistency between the predicted and the actual survival in both sets. Category-free NRI for 1, 3 and 5-year follow-up in the training set were 0.546 (95% CI: 0.376–0.655), 0.551 (95% CI: 0.368–0.679) and 0.468 (95% CI: 0.276–0.710), respectively, and 0.455 (95% CI: 0.307–0.610), 0.454 (95% CI: 0.305–0.615) and 0.356 (95% CI: 0.172–0.555), respectively, in the validation set. In addition, IDI for 1, 3 and 5-year follow-up in the training set were 0.082 (*P* < 0.001), 0.084 (*P* < 0.001) and 0.063 (*P* < 0.001), respectively, and 0.052 (*P* < 0.001), 0.057 (*P* < 0.001) and 0.048 (*P* < 0.001), respectively, in the validation set. The IPA estimates for the nomogram were greater than AJCC staging estimates in training set (19.6% vs. 7.8, 18.2% vs. 5.9 and 12.8% vs. 0.8%, for 1, 3 and 5-year IPA) and validation set (15.4% vs. 6.7, 15.9% vs. 5.7 and 9.1% vs. 2.9%, for 1, 3 and 5-year IPA). These results demonstrated the superior predictive power of the nomogram for OS over the AJCC staging system.

### Clinical use

**Fig. 4:**
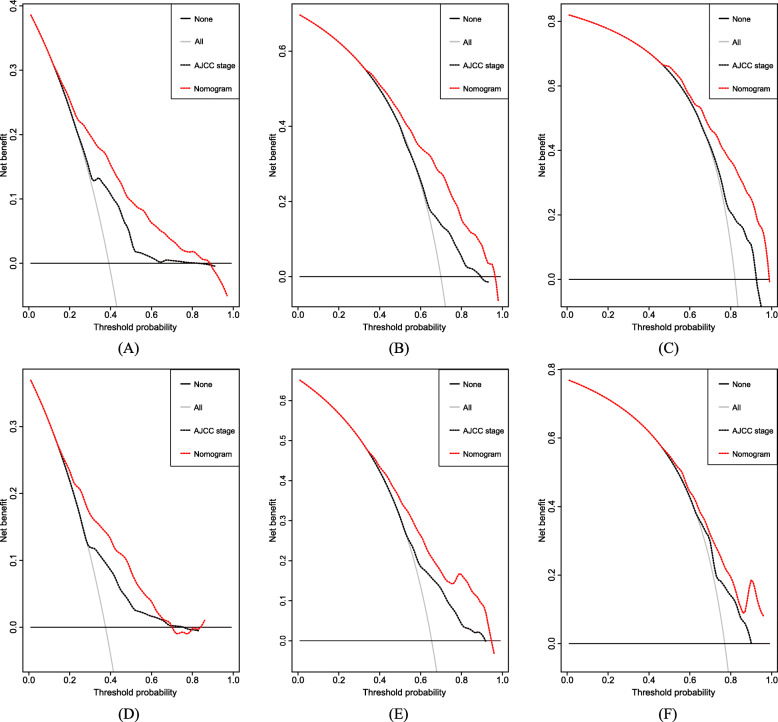
DCA for the nomogram and the AJCC staging system in the training set **a**, **b**, **c** and validation set **d**, **e**, **f** for 1, 3, and 5-year survival, respectively. Horizontal coordinates represented the threshold of probability, whereas the vertical coordinates represented the net benefit rate. The red and black dash lines represented DCA of the nomogram, and the AJCC staging system, respectively. The black solid line assumed all patients were alive, whereas the gray solid one with a negative slope assumed all patients were dead. DCA plot depicted the model with superior clinical application

DCA plots showed that our nomogram had greater net benefits in comparison with the AJCC staging system for predicting 1, 3 and 5-year OS of patients with metastatic RCC (Fig. 4), demonstrating its application in guiding clinical decision for metastatic RCC patients.

## Discussion

It is well accepted that clinical and pathological heterogeneity of metastatic RCC highly influences the prognosis of patients with the cancer [9]. Therefore, accurate prognosis prediction of the tumor continually frustrates efforts against the cancer. The IMDC model is the most widely used prognostic prediction model for metastatic RCC patients receiving targeted therapy. It is composed of six clinical and laboratory factors [8]. Using this model, patients can be stratified into three risk groups, each with different median OS. The efficacy of IMDC has been validated in metastatic RCC patients receiving first-line [8], second-line [21], and third-line targeted therapies [22]. Such models aside, risk-scoring systems including nomograms can also predict survival probability of individual patients. This study comprised of data for patients from multiple centers, archived in the SEER database. The focus was on the newly diagnosed metastatic RCC patients for whom the MSKCC and IMDC models might be unsuitable. Meanwhile, the predictive model proposed in this study is a nomogram, demonstrated to predict the OS more precisely. Apparently, accurate OS prediction is one of the most concerned issues for metastatic RCC patients. Our nomogram meets all the criteria necessary for endorsement by the AJCC [23], and has the potential to supplement the AJCC staging system.

With an HR of 1.54, lymph node metastasis negatively affected the survival of metastatic RCC patients. Using 2530 RCC patients treated with nephrectomy, Karakiewicz et al. constructed a nomogram for survival prediction based on six independent factors, including tumor stage, lymph node status, metastatic status, tumor size, Fuhrman grade, and type of symptoms [24]. Pati et al. also reported that lymph node metastasis strongly impacted on progression-free and overall survival of metastatic RCC patients [25], consistent with the findings at Cleveland Clinic [7]. Fuhrman grade, as the most widely accepted prognostic grading system, is not only associated with the survival of local RCC but also metastatic types [24]. In this study, compared with grade I, Fuhrman grade IV was associated with a worse OS after adjusting other clinical variables (HR = 1.80, 95% CI: 1.06–3.06, *P* = 0.029). Sarcomatoid differentiation is another predictor of short survival for metastatic RCC patients. Considering the small percentage of patients with sarcomatoid features (< 6%), sarcomatoid differentiation was not included in the IMDC model for OS prediction, though it is associated with adverse oncologic outcomes [8]. However, nearly 20% of patients included in this study exhibited sarcomatoid differentiation, with patients not displaying this feature shown to have superior OS (HR = 0.61, 95% CI: 0.50–0.75, *P* < 0.001). Therefore, it was included in our model.

Based on a Nationwide Inpatient epidemiologic study, lung was the most common metastatic site for metastatic RCC, accounting for 45.2% of such cases. Bone and liver metastases followed with 29.5 and 20.3%, respectively [26]. For this study, 61.7, 31.5, 11.1, and 9.9% of the patients in our cohort presented with lung, bone, liver, and brain metastases, respectively (Table 1). These four clinical variables were all significantly associated with adverse survival, and our findings were consistent with the growing evidence that the presence of distant metastasis predicted worse clinical outcomes. In one phase III trial of 375 metastatic CCRCC patients who received sunitinib, lung and liver metastases were significantly associated with poor progression-free survival [27]. In a separate study involving 281 metastatic RCC patients administered with third-line targeted therapy, it was demonstrated metastases at diagnosis were associated with adverse effects on survival [28]. In a separate retrospective study of over 2000 metastatic RCC patients who received first-line targeted therapy, bone or liver metastasis were independent risk factors for shorter OS. Intriguingly, compared with other metastatic sites, both bone and liver metastases were associated with worse survival. Also, the incorporation of bone and liver metastases significantly increased the predictive performance of the IMDC model [29].

In the era of cytokines, two randomized controlled trials demonstrated prolonged survival with nephrectomy plus interferon, relative to interferon alone among metastatic RCC patients [30, 31]. Early retrospective studies also corroborated the role of cytoreductive nephrectomy [32, 33]. However, the role of nephrectomy in treating metastatic RCC in the era of targeted therapy remains controversial. For instance, in one randomized trial (CARMENA), sunitinib monotherapy against intermediate- to poor-risk metastatic RCC was not inferior to a combination modality of nephrectomy followed by sunitinib [33]. Though the survival advantage to metastatic RCC patients with favorable characteristics was not evaluated in CARMENA, a significant proportion of metastatic RCC patients did not benefit from nephrectomy. Multiple retrospective studies investigated the association between nephrectomy and clinical outcomes among metastatic RCC patients receiving targeted therapy. Most of them supported this population could receive survival benefits from nephrectomy [34–37]. Using the IMDC database, Heng et al. reported metastatic RCC patients who received targeted therapy and cytoreductive nephrectomy had longer OS compared to those on targeted therapy alone (20.6 vs. 9.6 months, *P* < 0.001). After adjusting for other variables, the HR for death was 0.60 (95% CI: 0.52–0.69, *P* < 0.001) [34]. Comparable findings were reported following analysis of the National Cancer Data Base [35]. Most investigations analyzing the cytoreductive nephrectomy benefits are inherently limited by their retrospective nature. Notably, under any circumstances, patient selection and timing of surgery critically impact on nephrectomy benefits in metastatic RCC patients [33, 35]. Most often, patients with poor health status are unlikely to benefit from nephrectomy [38]. On the other side, patients with good performance status, minimal symptoms related to metastases, a resectable primary tumor, and a limited burden of metastatic disease are likely to highly benefit from the surgery [38]. One systematic review also found that rigorously selected patients based on prognostic factors, with less metastasis, and those displaying favorable responses to initial systemic therapy could highly benefit from nephrectomy [39]. In this study, patients recommended for surgery and who indeed underwent the procedure were more likely to benefit from nephrectomy relative to non operated patients. Subgroup analyses validated the robustness of the nomogram. It is noteworthy that there is no “one size fits all” approach for managing cancers [33]. Stated differently, the current findings do not recommend cytoreductive nephrectomy to all patients, but rather, clinicians should adopt rigorous selection criteria for surgery. The findings of this study are only based on retrospective data in the SEER database, thus should be interpreted with caution. As such, more investigations should be performed to strengthen the reliability of this model before its clinical application.

Our findings notwithstanding, this study suffered several limitations. First, because of the retrospective nature, inherent selection bias associated with such studies, in particular treatment selection bias was unavoidable. Because more relatively healthy patients were commonly recommended for surgery, it was more likely they would exhibit better clinical outcomes. Second, other independent RCC prognostic factors such as MSKCC or IMDC risk stratification, level of serum albumin, and lactate dehydrogenase were not captured in the SEER database. Third, some potentially valuable variables such as type of targeted therapy, timing of surgery, and other comorbidities were also not captured in the SEER database.

## Conclusions

In summary, we established a nomogram for survival prediction of metastatic RCC patients. It incorporated eight clinical factors: Fuhrman grade, lymph node status, sarcomatoid feature, cancer-directed surgery, bone, brain, liver, and lung metastases. The nomogram displayed superior predictive capability and higher clinical application than the conventional AJCC staging system.

## Data Availability

The datasets generated and/or analyzed during the current study are available in the SEER repository (http://www.seer.cancer.gov).

## Abbreviations

OS: Overall survival
RCC: Renal cell carcinoma
SEER: Surveillance, Epidemiology and End Results
C-index: Harrell’s concordance index
IDI: Integrated discrimination improvement
NRI: Net reclassification improvement
IPA: Index of prediction accuracy
DCA: Decision curve analysis
AJCC: American Joint Committee on Cancer
MSKCC: Memorial Sloan-Kettering Cancer Center
IMDC: International Metastatic RCC Database Consortium
CCRCC: Clear cell renal cell carcinoma
PRCC: Papillary renal cell carcinoma
CHRCC: Chromophobe renal cell carcinoma
SRCC: Sarcomatoid renal cell carcinoma
CDRCC: Collecting duct renal cell carcinoma
HR: Hazard ratio
95% CI: 95% confidence interval
